# Long-term warming modulates diversity, vertical structuring of microbial communities, and sulfate reduction in coastal Baltic Sea sediments

**DOI:** 10.3389/fmicb.2023.1099445

**Published:** 2023-03-29

**Authors:** Laura Seidel, Varvara Sachpazidou, Marcelo Ketzer, Samuel Hylander, Anders Forsman, Mark Dopson

**Affiliations:** Centre for Ecology and Evolution in Microbial Model Systems (EEMiS), Linnaeus University, Kalmar, Sweden

**Keywords:** climate change, sediments, 16S rRNA gene amplicon, depth profile, microbial communities

## Abstract

Coastal waters such as those found in the Baltic Sea already suffer from anthropogenic related problems including increased algal blooming and hypoxia while ongoing and future climate change will likely worsen these effects. Microbial communities in sediments play a crucial role in the marine energy- and nutrient cycling, and how they are affected by climate change and shape the environment in the future is of great interest. The aims of this study were to investigate potential effects of prolonged warming on microbial community composition and nutrient cycling including sulfate reduction in surface (∼0.5 cm) to deeper sediments (∼ 24 cm). To investigate this, 16S rRNA gene amplicon sequencing was performed, and sulfate concentrations were measured and compared between sediments in a heated bay (which has been used as a cooling water outlet from a nearby nuclear power plant for approximately 50 years) and a nearby but unaffected control bay. The results showed variation in overall microbial diversity according to sediment depth and higher sulfate flux in the heated bay compared to the control bay. A difference in vertical community structure reflected increased relative abundances of sulfur oxidizing- and sulfate reducing bacteria along with a higher proportion of archaea, such as Bathyarchaeota, in the heated compared to the control bay. This was particularly evident closer to the sediment surface, indicating a compression of geochemical zones in the heated bay. These results corroborate findings in previous studies and additionally point to an amplified effect of prolonged warming deeper in the sediment, which could result in elevated concentrations of toxic compounds and greenhouse gases closer to the sediment surface.

## 1.Introduction

Climate change is and will continue to challenge all living organisms with interactions between many factors in the environment the organisms inhabit. This will modulate how species, communities, and the ecosystem services that they provide respond to climate change. Coastal water areas, such as those in the Baltic Sea, already suffer from anthropogenic nutrient input resulting in accelerated eutrophication (Malone and Newton, 2020) and these type of systems are globally also influenced by climate related effects such as acidification, altered salinity, stratification, sea level rise, higher river runoff, and increased temperatures (Bindoff et al., 2019). Several of these effects will lead to further changes interwoven within each other, such as higher metabolic rates of microorganisms due to increased temperatures (Clarke and Fraser, 2004). This will result in amongst other effects, enhanced oxygen consumption (Breitburg et al., 2018; Hicks et al., 2018) at the sediment-water interface where organic matter (OM) is mineralized (Burdige, 2006).

The Baltic Sea is a semi-enclosed water body with limited exchange to the open sea (Kullenberg and Jacobsen, 1981). Climate change already shows a strong warming trend within the Baltic Sea with a sea surface temperature increase of 0.6°C per decade between 1990–2008 (ICES, 2019). Coastal ecosystems are some of the most productive but are highly sensitive to anthropogenic factors (Bini and Rossi, 2021). Climate change will challenge this ecosystem by potential accelerated eutrophication effects resulting in the expansion of coastal hypoxia (Reusch et al., 2018), but also higher precipitation rates leading to increased nutrient loads due to higher river-run off (IPCC, 2019). Altieri and Gedan (2015) demonstrated that alterations driven by climate change, such as temperature, acidification, and wind and storm patterns strengthen the impact of hypoxia. These changes will be even greater in coastal zones (Gilbert et al., 2010), emphasizing the need to understand how changes in the future will alter microbial communities and their nutrient cycling at the base of the food web.

Microbial communities in marine sediments play a key role in the biogeochemical cycling of, e.g., carbon, nitrogen, and oxygen (Madsen, 2011; Broman et al., 2017, 2018). For example, Hicks et al. (2018) show that microbial communities within coastal sediments change their composition and activity with increasing temperature that in turn, also affects nutrient availability and will drive future changes in marine biogeochemical cycling. Within coastal benthic waters, higher temperatures will likely lead to a decrease in bacterial diversity (Seidel et al., 2022a). In contrast, higher oxygen consumption due to various factors such as temperature and eutrophication compresses the redox layers within sediments (Mahmoudi et al., 2014) resulting in increased bacterial diversity (Seidel et al., 2022b). As a result, oxygen will be completely consumed closer to the sediment-water interface and alternative electron acceptors, such as NO_3_^–^, Mn^2+^, Fe^2+^, SO_4_^2–^, and CO_2_ are used to further metabolize the OM (Burdige, 2006). Sulfate reduction reduces sulfate to toxic sulfide, which can be directly re-oxidized chemically or by sulfur oxidizing microbes (Burdige, 2006; Broman et al., 2017). However, it is unknown if the altered microbial community composition and vertical compression of the geochemical zones of the redox-potential within surface sediments (Seidel et al., 2022b) will result in the release of these greenhouse gases (e.g., CO_2_ and CH_4_) and toxic compounds (e.g., S_2_^–^) to the water column, respectively. It is further unknown if the condensation of the geochemical zones will decrease the efficiency of the microbial filter (*via* sulfate reduction) that prevents methane diffusing from below to reach the water column and eventually the atmosphere.

This study was executed in two Baltic Sea bays. One that has been used as a cooling water outlet for a nearby nuclear power plant and therefore, provided an ideal environment to study the effect of increased temperature [while maintaining otherwise natural yearly cycles (Seidel et al., 2022b)] to those predicted within the RCP5-8.5 scenario (IPCC, 2021), which may occur in coastal areas under less extreme predictions. The other was an unaffected nearby bay with similar surrounding conditions. Previous studies in this experimental system show the effects of long-term warming on bottom water and surface sediment microbial communities, demonstrating an increased relative abundance of Picocyanobacteria in the heated bay in summer months and decreased overall bacterial alpha diversity (Seidel et al., 2022a). In addition, there was an increase in alpha diversity in the heated bay surface sediments and a shift of the microbial community toward the seafloor resulting in the thinning of the geochemical zones (Seidel et al., 2022b). However, it has not yet been investigated how bacterial communities underneath the sediment surface at lower depths are affected by long-term warming.

The aims of this study were to investigate whether and how prolonged warming affects the composition and vertical structuring of microbial communities and if such effects would affect sulfate fluxes in bottom compared to surface sediments. It was hypothesized that the prolonged increased temperature will influence microbial alpha-diversity as well as resulting in an altered microbial community with higher sulfate reduction rates along the depth gradient. The specific questions addressed were: how do the microbial diversities change with depth in a long-term heated bay compared to an unaffected control bay; how does the microbial composition along the depth gradient change after long-term heated for 50 years; and how does this effect sulfate cycling and how does this inform for future warming scenarios?

## 2. Materials and methods

### 2.1. Study system–Description of the heated and control bays

The underlying study was conducted on 18 and 19 June 2018 at two coastal Baltic Sea bays (GPS coordinates can be found in Supplementary Table 1) north of the city of Oskarshamn, Sweden. One bay has been used as a cooling outlet for a nearby nuclear power plant for the last 50 years that increased the temperature on average around 5°C compared to the ambient bay. The second bay was a control bay, which was not connected to the artificially heated bay beside the open Baltic Sea with approximately 1.5 km distance. The control bay was chosen as it was as close as possible to the heated bay without being influenced by it. The two bays have been used in previous studies with further information therein (Seidel et al., 2022a,b).

### 2.2. Monitoring and sampling of the Baltic Sea bays

Temperature monitoring was conducted at three points in each bay according to Seidel et al. (2022a). Briefly, temperature was measured every 2 h at 1 m below surface as well as bottom water temperature of each sampling site during core retrieval (Supplementary Figure 1). Three sampling sites per bay (Supplementary Figure 2) were selected and three sediment cores per site were sampled using a Kajak gravity corer with a transparent liner (inner diameter: 7 cm, length: 60 cm) as described in Seidel et al. (2022a). Briefly, the cores were sliced and sampled at average sediment depths of 0.5 cm (surface), 7 cm (middle), and 24 cm (deep) for 16S rRNA gene amplicon sequencing (Supplementary Table 1). The surface (Seidel et al., 2022a,b) as well as the control bay middle and deep (Westmeijer et al., 2022) samples have been previously published. Additionally, 15 mL of sediment was collected at the same depths (surface, middle, and deep) for sulfate (SO_4_^2–^) concentration analysis in porewaters as measured *via* spectrophotometry using the Hach-Lange cuvette test LCK353 (Supplementary Table 1). Sulfate diffusive fluxes for the sampling time point between the surface and deep samples were calculated using Fick’s first law and the effect of porosity on diffusion was considered through a logarithmic equation (Boudreau, 1996). The average temperature measured in each bay and assumed salinity of 6 psu and sediment porosity of 0.8 (within the range of uncompacted mud; Mondol et al., 2007) was used.

### 2.3. DNA extraction and sequencing

DNA extraction and sequencing was conducted according to Seidel et al. (2022a),Seidel et al. (2022b) and briefly, sediment DNA extraction was performed using the DNeasy^®^ PowerSoil Extraction Kit (QIAGEN, Germany) according to the manufacturer’s guidelines. 16S rRNA gene amplification was performed using the PCR primers 341f and 805r (Hugerth et al., 2014) and the library for sequencing was prepared and sent for sequencing to the Science for Life laboratory (SciLifeLab) in Stockholm, Sweden. The raw sequencing data were trimmed, denoised, merged, and chimeras removed using the nf-core ampliseq (v. 2.4.0dev) pipeline built in Nextflow (v. 22.04.4) (Straub et al., 2020) running within the UPPMAX cluster (Uppsala Multidisciplinary Center for Advanced Computational Science). The sequences were trimmed at 269 bp forward and 209 bp reverse and the settings—-double primer were set to “true” to run cutadapt (v. 3.4) twice to ensure the removal of primers, as well as the—-sample_interference were set to “independent.” After chimera removal, the taxonomy was assigned to the SBDI-GTDB (Sativa curated 16S GTDB database, R06-RS202-1, FigShare. doi: 10.17044/scilifelab.14869077.v3). Further data analysis was conducted using R (v. 4.0.4) (R Core Team, 2018).

### 2.4. Bioinformatics and statistical analysis of the 16S rRNA gene amplicon data

Analysis of the data was according to (Seidel et al., 2022a,b). Rarefaction curves were calculated (Supplementary Figure 3) and unknown cyanobacterial sequences annotated as eukaryotic diatoms were removed before further analysis. Alpha diversity was calculated (Shannon’s H Index) and significant differences between the data were tested using a linear mixed model. First a model with bay and sediment depth, as well as the interaction between bay and sediment depth, were used as fixed categorical variables, while sampling site was included as a random factor. No significance was observed for the interaction between bay and sediment depth, so a simpler model without the interaction was used to evaluate the effects of sediment depth and bay. Furthermore, to compare the bays at each depth, a pairwise comparison was performed (Supplementary Table 2) and the *p*-values were adjusted (Bonferroni correction method). Sulfate concentrations in the different bays and depths were tested using a linear mixed model with bay, depth, and the interaction between bay and depth as fixed categorical variables, while sampling site was included as a random variable (Supplementary Table 2). Finally, to compare the different depths within bays, a pairwise comparison was performed, and the *p*-values were adjusted using the same method as previously described. To test for dissimilarities in the microbial communities, a non-metric multidimensional scaling (nMDS) was performed based on Bray–Curtis dissimilarities using relative abundances. Differential abundance analysis was performed based on the summarized counts on class level (filter of rare taxa, *n* = 233 taxa retained; Supplementary Table 3). The model used bay and depth as interaction and the contrast function was used to compare the bays at each depth, as well as the different depths within the bays. Finally, a PICRUSt2 analysis on the 16S rRNA gene amplicon sequences (ASVs) was conducted according to the workflow guidelines^1^ (Douglas et al., 2020). Kyoto Encyclopedia of Genes and Genomes (KEGG) based KEGG Orthology (KO) identification numbers related with sulfur cycling were filtered and relative abundances were calculated based on depth and coastal bay to generate a Bray Curtis distance-based RDA (db-RDA) using the vegan package (version 2.5-7). Predicted functions associated with dissimilatory sulfate reduction und sulfide oxidation were selected and a bar plot generated.

## 3. Results

Sediment samples at three different depths were collected in mid-June 2018 within a Baltic Sea “heated” and a “control” bay at three sampling sites for each bay (Supplementary Figure 2; Supplementary Table 1). The bottom water temperature of the heated bay was on average 21°C in June 2018 while it was 13°C in the control bay (Seidel et al., 2022b).

### 3.1. Pore water sulfate concentrations between bays at the three different depths and sulfate fluxes

**FIGURE 1:**
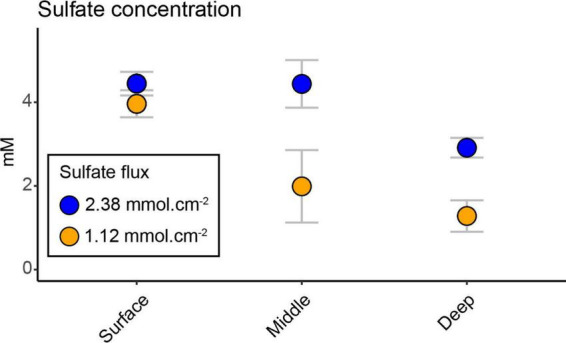
Sulfate concentrations; mean (*n* = 3) ± s.d. sulfate concentration in the heated and control bays at the three different depths. Sulfate fluxes for each bay were calculated based on average temperature measured in each bay and assumed salinity of 6 psu and sediment porosity of 0.8 (within the range of uncompacted mud).

The sulfate concentration showed a significant decrease between the two bays with depth (interaction effect of bay and depth, *F*_2,20_ = 19.69, *p* < 0.0001; Supplementary Table 2) and had its lowest concentration at the deepest sediment (mean ± s.d. 1.27 ± 0.37 mM heated bay vs. 2.91 ± 0.23 mM control bay; Figure 1). The heated bay sulfate concentration at the middle and deep depths were significantly lower compared to the control bay (pairwise comparison heated vs. control bay, middle *p* < 0.0001 and deep *p* < 0.0001; Figure 1 and Supplementary Table 2). However, there were no significant differences between the surface (4.44 ± 0.28 mM) and middle (4.43 ± 0.56 mM) sediments in the control bay, while there was a significant drop in the deepest sediment (2.91 ± 0.23 mM, pairwise comparison, middle vs. deep, *p* = 0.0005; Supplementary Table 2). A different pattern was observed in the heated bay where sulfate concentrations significantly decreased at, e.g., the surface (3.96 ± 0.32 mM) vs. middle (1.98 ± 0.86 mM; pairwise comparison, *p* < 0.0001; Supplementary Table 2). The calculated average sulfate flux for the heated bay (2.38 mmol.cm^–2^) was approximately twice the control bay rate (1.12 mmol.cm^–2^) at the time of sampling.

### 3.2. Microbial diversity between the heated and control bay at different depth

**FIGURE 2:**
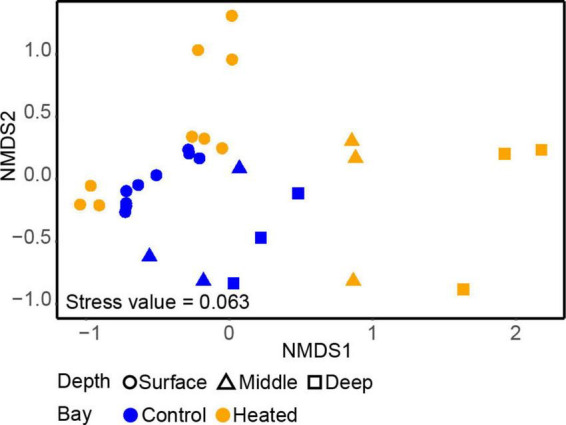
Non-metric multidimensional scaling (nMDS) of the samples within the heated (orange) and control (blue) bays at the three different sediment depths.

The alpha diversity based on the Shannon’s H index comparing microbial communities between the different depths in each bay showed that depth (but not bay) plays a significant role, with diversity tending to be lowest at intermediate section and highest at the deepest section of the sediment (Supplementary Figure 4 and Supplementary Table 2, ANOVA, effect of depth, *F*_2,20_ = 3.74, *p* = 0.04). A difference in vertical structure between the two bays was also observed in the beta-diversity based on the nMDS used to compare the 16S rRNA gene amplicon sequencing data of the two communities (Figure 2). There was a clear separation between the different depths in both bays on the first axis, although the vertical differences in the control bay were not as distinct as in the heated bay (Figure 2). Surface bacterial communities differed less between the bays at similar sediment depths compared to the deeper sampled sediments. On the second axis, the communities were more distinct, showing strong clustering toward specific sampling sites within the bay. In contrast, the control bay clustered on all depths closer to the surface samples, while the heated bay had three clear distinct clusters for each depth on the first axis and differences between the sampling sites on the second axis (Figure 2).

### 3.3. 16S rRNA amplicon based microbial community differences between the heated and control bays

**FIGURE 3:**
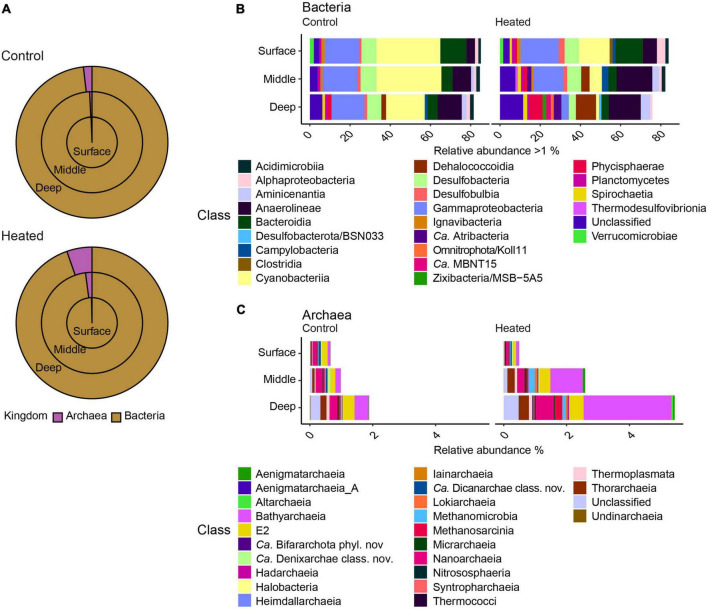
Relative abundances of bacteria and archaea from the two bays at the three sediment sampling depths showing the surface (outer circle), middle (middle circle), and deep (inner circle) communities **(A)** along with stacked bar graphs of bacteria **(B)** with >1% relative abundance and archaea **(C)** on the class level for the different sampling depths in the two bays.

The microbial community was dominated by bacteria (Figure 3) and the highest relative abundance on class level in both bays were Gammaproteobacteria, which showed similar relative abundances at all depths (12.48 ± 3.4% heated bay vs. 16.90 ± 3.1% control bay; relative abundance calculated per sample and summarized to mean by bay) except for the deepest sample in the heated bay (3.69 ± 0.4%). The highest relative abundances found in the control bay were Cyanobacteria (31.74 ± 13.3% mean per bay surface to 19.25 ± 20.8% deep), followed by Anaerolineae (4.31 ± 1.2% to 11.98 ± 2.1) that increased with depth while Bacteroidia decreased (12.93 ± 2.5% to 5.00 ± 2.0; Figure 3). The heated bay relative abundances of Cyanobacteria were 52% lower at the sediment surface compared to the control bay and decreased by 91% with depth (15.28 ± 11.0% surface to 1.45 ± 0.5% deep, heated bay), while the classes Anaerolineae (7.00 ± 2.8% to 15.86 ± 9.0%), Dehalococcodia (0.14 ± 0.09% to 10.00 ± 4.4%), and Phycisphaerae (1.01 ± 0.3% to 6.39 ± 1.2%) increased with depth (Figure 3).

The relative abundance of archaea increased with greater depth from around 0.3–0.6% in the surface sample to 1.8 and 5.3% (relative abundance calculated per sample and summarized to mean by bay) in the deep samples from the control and heated bays, respectively (Figure 3). The main archaea classes responsible for the changes in relative abundances between the different depths were Bathyarchaeia (mean ± s.d. relative abundance within each bay, 1.30 ± 1.3% heated bay vs. 0.23 ± 0.1% control bay), Nanoarchaeia (0.29 ± 0.1% vs. 0.20 ± 0.1%) in both bays, and Methanosarcinia (0.11 ± 0.1%) in the heated bay (Figure 3).

Finally, taxa involved in sulfur cycling were found at higher abundances in the heated bay compared to the control bay at various depths (Figures 3B, C). These included for example the sulfate reducing bacteria Desulfobulbia (surface relative abundance per bay and depth 3.0% ± 1.3 heated vs. 1.25% ± 0.6 control and middle 1.9% ± 1.0 heated vs. 1.5% ± 0.5 control) or the taxa Desulfobacterota/BSN033 (deep 1.2% ± 0.1 heated) and Zixibacteria/MSB-5A5 (deep 1.8% ± 1.1 heated), which were only found in higher relative abundances in the deep sediment of the heated bay. Furthermore, sulfate reducing archaea such as Bathyarchaeia (middle 1.0% ± 0.6 vs. 0.2% ± 0.1%, deep 2.8% ± 1.8% vs. 0.4% ± 0.1) as well as Thorarchaeia (middle 0.2% ± 0.09 vs. 0.07% ± 0.004, deep 0.3% ± 0.08 vs. 0.1% ± 0.1%) were found in higher relative abundance in the heated bay middle and deep sediment depths. This pattern was especially present within archaea in the heated bay. Finally, the relative abundance of sulfate reducers was higher at all depths in the warm compared to the control bay where the relative abundance only increased significantly in the deepest section (Figures 3B, C).

### 3.4. Differentially abundant ASVs with LFC > 2 on family level between the bays at the three depths

**FIGURE 4:**
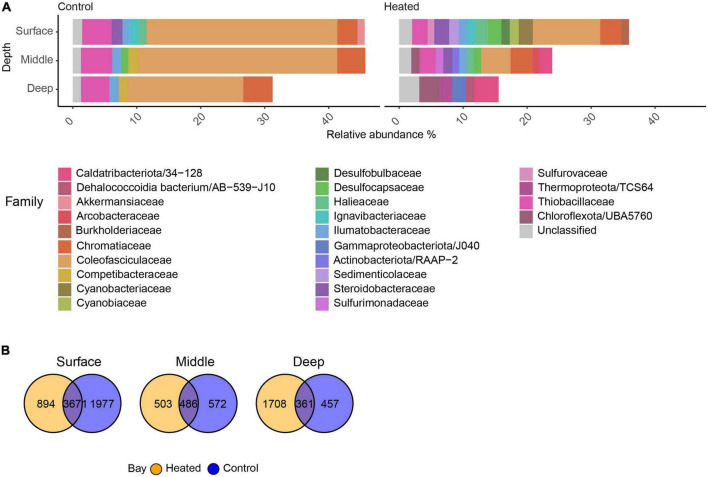
Significant differential abundant families with relative abundance above 1% and a log_2_ fold change of at least two within the heated and control bay at the three different depths **(A)** and venn diagrams of the significant ASVs within the heated and control bays at the three sediment depths **(B)**.

The highest significantly differential abundant ASVs with a log_2_ fold change (LFC) > 2 showed that within Cyanobacteria, the family Coleofasciculaceae showed high relative abundance in the control bay at all sediment depths (26.28 ± 17.0%) compared to the heated bay where its highest relative abundance was found in the surface (10.51 ± 8.8%) and decreased rapidly with depth (deep, 0.64 ± 0.1; Figure 4). In general, fewer ASVs were associated with the highest differential abundant taxa in the control bay, such as Thiobacillaceae that were abundant in all three depths (4.63 ± 2.1%). In contrast, the heated bay showed large differences between the different depths compared to the control bay including high relative abundances of 16S rRNA gene amplicons aligning within the classes Desulfobulbaceae, Desulfocapsaceae, Sedimenticolaceae, Sulfurimonadaceae, and Sulfurovaceae in the surface and middle sediment (Figure 4). Within the top differential abundant taxa, the number of unique ASVs decreased in the control bay with depth with 1,977 unique ASVs in the surface followed by the 572 and 457 ASVs in the middle and deepest sections, respectively (Figure 4). The shared ASVs between both bays also decreased with depth while the heated bay surface sediment had 894 unique ASVs dropping to 572 in the middle and increasing again at the deeper sediments.

### 3.5. Sulfur cycling related functional abundances

**FIGURE 5:**
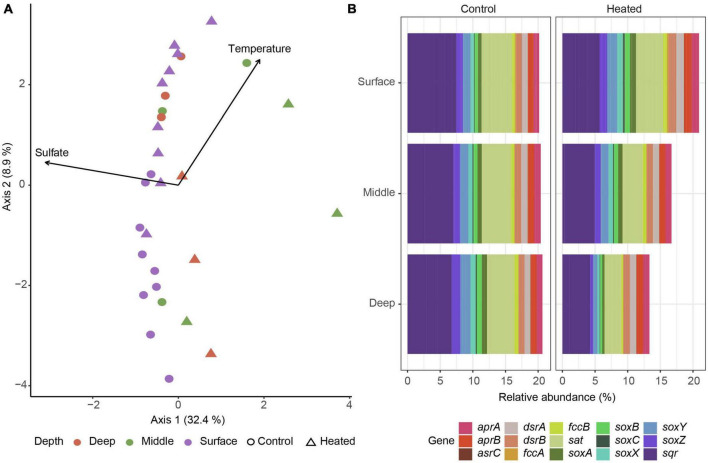
16S rRNA gene ASV based prediction of sulfur cycling related functional abundances. Bray–Curtis based db-RDA of predicted functional genes related with sulfur cycling according to KEGG identifiers for the heated (triangle) and control (circle) bays at surface (purple), middle (green), and deep (red) sediment depths with the environmental parameters of sulfate and temperature **(A)** along with plots of relative abundances of selected dissimilatory sulfate reduction and sulfide oxidation genes in the control (left) and heated (right) bay **(B)**.

16S rRNA gene based functional predictions of sulfur cycling related genes showed a general trend of middle depth samples splitting from the other depths on the first axis explained by both temperature and sulfate while sediment depth and temperature were distinguishing factors on the second axis with heated bay surface gene predictions mixed with control bay middle and deep samples (Figure 5A). Analysis of specific genes related to dissimilatory sulfate reduction, e.g., *dsrAB* and inorganic sulfur compound oxidation such as the *sox* genes showed little differences in the control bay compared to a reduced relative abundance of predicted sulfur oxidation genes with depth in the heated bay (Figure 5B).

## 4. Discussion

Prolonged and increased warming will alter future coastal environments, although the degree that microbial communities and their energy- and nutrient cycling will change and how this will affect trophic levels within the marine food web are largely unknown. The main focus of this study was to investigate how changes in the microbial community at different sediment depths were affected by warming and how likely this will result in increased concentrations of toxic compounds within sediments.

The variation in microbial diversity at the different depths were interwoven with the contrasting microbial community composition, showing alterations within the different depths of the heated bay sediment with for example, increased sulfate reducing bacteria (SRB) and elevated sulfate reduction compared to the control bay. This was supported by the 16S rRNA gene amplicon-based prediction of aerobic sulfur oxidation genes that decreased more rapidly with depth in the heated versus control bay. In addition, Cyanobacteria are maintained in the marine deep biosphere *via* organic carbon degradation (Puente-Sánchez et al., 2018) and the presence of the Coleofasciculaceae family at all investigated control bay depths compared to the decrease in the heated bay with depth suggested more rapid organic carbon degradation. Hicks et al. (2018) show that increased temperature results in community composition change (including an increase in SRB) potentially due to higher use of various electron acceptors and therefore driving, e.g., sulfate reduction toward the sediment surface (Mahmoudi et al., 2014). Such thinning of the redox layer closer to the sediment surface, as well as the increase in microbial diversity, is also shown in previous studies of our system (Seidel et al., 2022a,b). These observations corroborate the idea of condensation of the geochemical zones revealed by the sulfate measurements in pore water, i.e., that sulfate is consumed faster and at shallower depths in the warm bay resulting in a thinner sulfate reduction zone. The calculated higher sulfate flux in the warm bay further corroborates this idea. This thinner sulfate reduction zone may also suggest that upward diffusing methane will have a higher chance to bypass this microbial filter and reach the water and eventually the atmosphere.

Studies by Hinrichs and Boetius (2003) suggest a syntrophic relationship between SRBs and archaea potentially linking the significantly more sulfate reducing and sulfide oxidizing bacteria (SOB) within surface and middle depth of the heated bay to the higher relative abundance of Bathyarcheota. The SRBs and SOBs were only found in higher relative abundances in the deep section of the control bay that was consistent with the potential compression of redox layers and increased microbial diversity in the shallower sediments. These included SOB family members Sulfurimonadaceae and Sulfurovaceae as well as the SRBs members such as Desulfobulbaceae, Desulfocapsaceae, and Sedimenticolaceae often associated with anoxic sediments (Orcutt et al., 2011). For example, within the SOB, *Sulfurovum* were found in higher relative abundance in the surface and middle layers in the heated bay and only showed higher relative abundances in the deeper section in the control bay. A similar pattern was observed within the SRB families such as Desulfobulbaceae and Desulfocapsaceae as well as the genera *Desulfobulbus*, *Desulfofustis*, *Desulfopila*, and *Desulforhopalus*. Overall, the increased diversity of SRB in the long-term heated sediment, especially in the surface and middle sections, could also be potentially linked to the higher sulfate reduction, which dropped more rapidly with depth compared to the control bay and was generally significantly lower in the heated bay. Robador et al. (2016) show that SRB mediated sulfate reduction is controlled by temperature and that SRB communities inhabiting geographically distinct environments with lower temperatures, such as the Arctic and Antarctic, are more similar than communities in more proximate warmer locations (e.g., Arabian Sea and southern North Sea). In contrast, an earlier study by Robador et al. (2009) indicates that seasonally changing temperate zone SRB abundances and sulfate reduction rates show only limited response to changing temperature after 24 months of incubation. However, the incubation temperatures used within the study by Robador et al. (2009) were below the average temperature of the heated bay and did not reflect the temperature span of seasonal changes within potential long-term warming projections for coastal temperate zones such as the Baltic Sea (Reusch et al., 2018; ICES, 2019).

The higher relative abundance in the heated bay also included increased relative abundance of especially Bathyarchaea with increasing depth. While the primer sets used for the analysis of the 16S rRNA gene sequences are biased toward bacteria (Hugerth et al., 2014), the identified archaea showed distinct differences between the two bays. Archaea are widely found in marine sediments and the class Bathyarchaea, belonging to the phylum Thermoproteota and previously known as Crenarchaea (Oren and Garrity, 2021), are one of the predominant groups within the marine subsurface archaeal community (Kubo et al., 2012; Lloyd et al., 2013). The higher relative abundance of archaea in the long-term heated bay, especially with increasing depth could be linked to the potential involvement of Bathyarchaea in methane metabolism (Lloyd, 2015) as well as their correlation of higher relative abundances with increasing depth (Zhou et al., 2018) and within anoxic environments (Fillol et al., 2016). On the family level, higher relative abundances of methanogenic BA-1 (Evans et al., 2015), such as Bathy-8 were found in the heated bay, fitting to the preference of these archaea for deeper sediments (Lazar et al., 2015). Another study from Aarhus Bay (Denmark) sediments showed that Bathyarcheota are found in higher abundance in sulfate-methane transition zones (Webster et al., 2011), which likely was the case for the increase at the different depths of the heated bay sediments. Additionally, more diverse subgroups of Bathyarcheota were found in the heated bay on all depth layers, potentially influencing the higher diversity and greater distinction within heated bay sediment layers. Those increased relative abundances of methanogens as consequences of the shallowing of the geochemical zones could potentially lead to elevated production of methane closer to the seafloor and therefore, increase the likelihood of methane reaching the water column and perhaps the atmosphere.

Future climate change projections on how prolonged and elevated warming will affect coastal marine ecosystems rely on many assumptions. This study suggested sediment microbial communities will alter due to, e.g., a greater prevalence of oxygen deficient zones and increased respiration rates with higher amount of OM will likely result in higher numbers and greater diversity of microbial communities. The increase in OM decomposition could potentially lead to a chain reaction, with likely increased sulfate reduction rates along the sediment depth gradient. In contrast, studies of sulfate reduction rates by microbial communities within Arctic, temperate, and tropical marine sediment show that increasing temperature will likely not result in generally higher carbon mineralization rates, as microbial organisms optimize their metabolic rates under long-term ambient temperatures (Robador et al., 2016). Nevertheless, the various factors that are modified by rising temperatures, for example due to climate change, result in altered microbial communities. Additionally, rising temperatures will likely lead to higher sulfate reduction rates and potentially higher generation of toxic compounds, as has been suggested in previous studies (Seidel et al., 2022b).

## Data availability statement

The 16S rRNA gene sequencing data are available on the NCBI database under BioProject PRJNA739524 and PRJNA901918, as well as ENA under the project number PRJEB41312. The code to generate the figures and statistical testing can be found on https://github.com/laseab/3depth_comp.

## Author contributions

LS, MK, SH, AF, and MD designed the study. LS, MK, and VS collected and processed the data. LS analyzed the data and carried out the statistical approach. LS and MD drafted the manuscript. All authors read and approved the final manuscript version.

## Footnotes

1
github.com/picrust/picrust2/wiki
