# Supplementary material for: Long-term warming modulates diversity, vertical structuring of microbial communities, and sulfate reduction in coastal Baltic Sea sediments
Source: Front Microbiol. 2023 Mar 29;14:1099445. doi: 10.3389/fmicb.2023.1099445 (PMC10090409; doi:10.3389/fmicb.2023.1099445)
Supplement: Supplementary file 1 [file Data_Sheet_1.docx]

Supplementary Material

**Long-term warming modulates diversity, vertical structuring of microbial communities and sulfate reduction in coastal Baltic Sea sediments**

Laura Seidel, Varvara Sachpazidou, Marcelo Ketzer, Samuel Hylander, Anders Forsman, and
Mark Dopson

**Supplemental Table 1**

A separate Excel file has been uploaded.

**Supplemental Table 2.**

A separate Excel file has been uploaded.

**Supplemental Table 3.**

A separate Excel file has been uploaded.

# Data Availability Statement

The 16S rRNA gene sequencing data are available on the NCBI database under BioProject PRJNA739524 & PRJNA901918, as well as ENA under the project number PRJEB41312. The code to generate the figures and statistical testing can be found on https://github.com/laseab/3depth_comp.


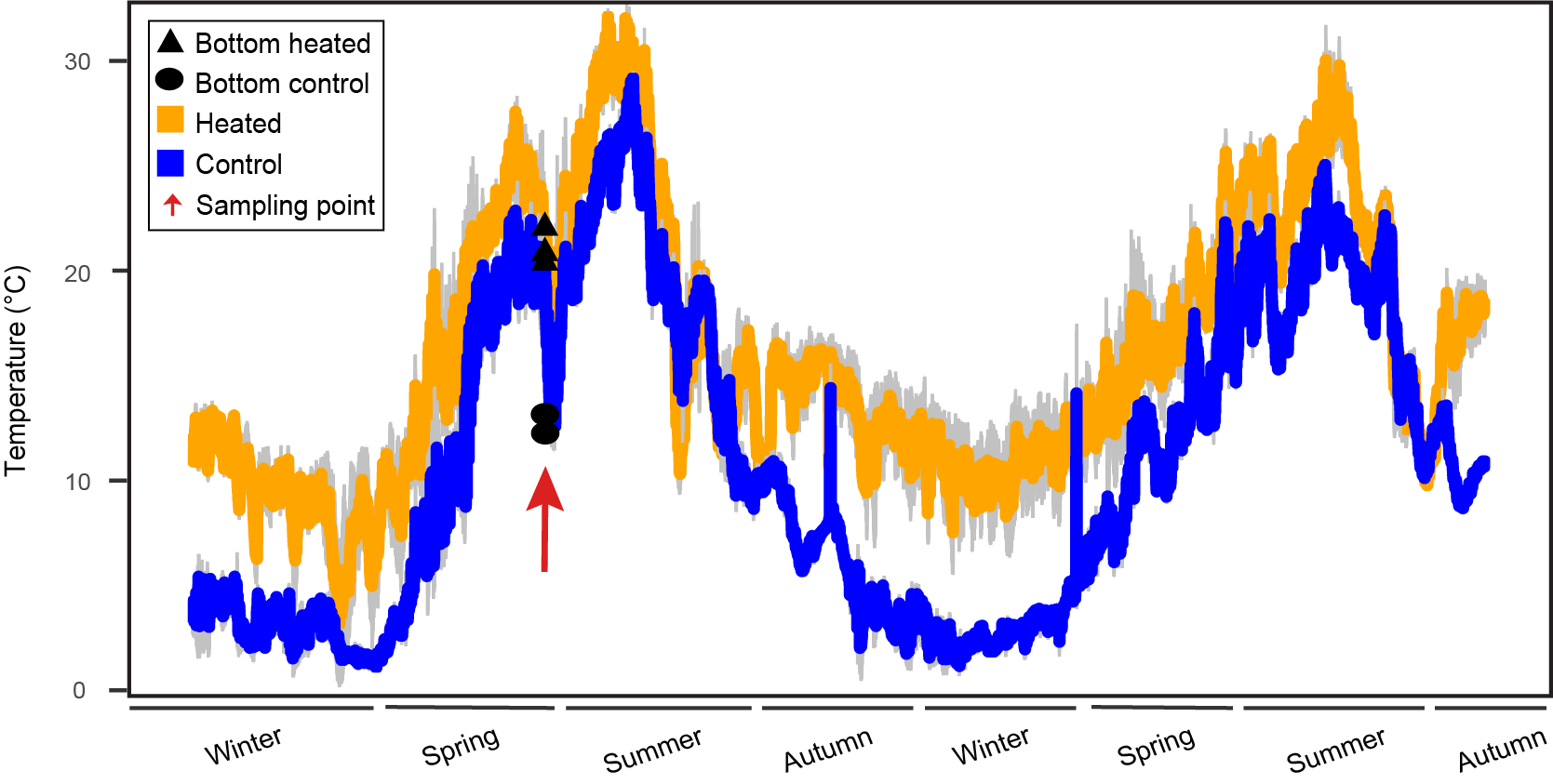


Figure S1. Temperature tracking of the heated and control bays. Included are the measured temperatures in bottom water of the heated (black triangle) and control (black circle) and the sampling point (red arrow); Figure adapted from Seidel et al. (2022) Weakened resilience of benthic microbial communities in the face of climate change. ISME Communications 2(1), 21. doi: 10.1038/s43705-022-00104-9.


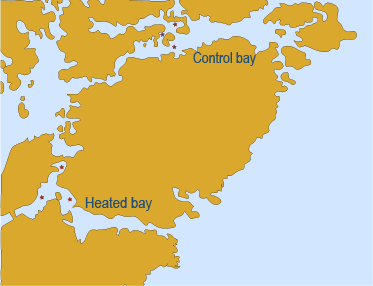


Figure S2. Map including sampling sites**.**


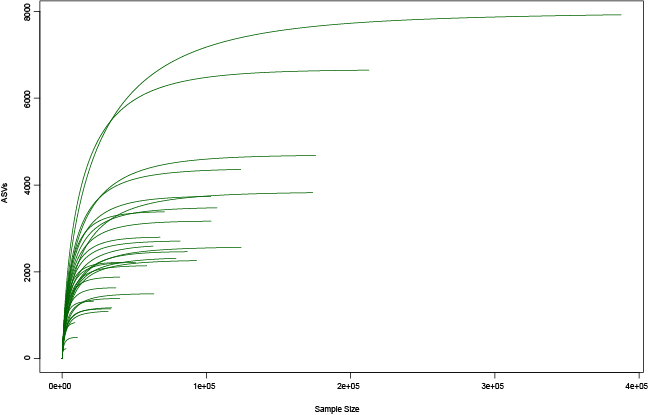


Figure S3. Rarefaction curves of the sequenced samples.


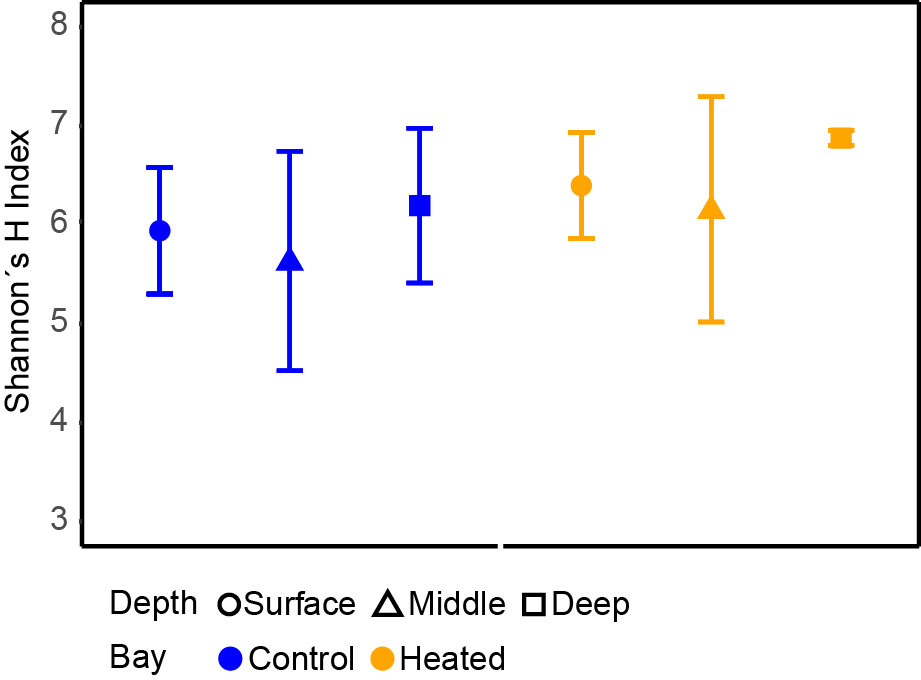


Figure S4. Shannon´s H index alpha diversity from the heated and control bay at the three different depth. Shown are the mean ± s.d. (all *n*=3) for the heated and control bays.
